# The assessment of future RSV immunizations: How to protect all infants?

**DOI:** 10.3389/fped.2022.981741

**Published:** 2022-08-09

**Authors:** Louis Bont, Catherine Weil Olivier, Egbert Herting, Susanna Esposito, Jose Antonio Navarro Alonso, Federico Lega, Silke Mader, Ichiro Morioka, Kunling Shen, George A. Syrogiannopoulos, Saul N. Faust, Elena Bozzola

**Affiliations:** ^1^Department of Paediatrics, Wilhelmina Children's Hospital, University Medical Centre Utrecht, Utrecht, Netherlands; ^2^Department of Pediatrics, Paris 7 University, Paris, France; ^3^Department of Pediatrics, University of Lübeck, Lübeck, Germany; ^4^Department of Medicine and Surgery, Pediatric Clinic, Pietro Barilla Children's Hospital, University of Parma, Parma, Italy; ^5^Deparment of Vaccinology, Ministry of Health, Madrid, Spain; ^6^Department of Biomedical Science, Research Center in Health Administration, University of Milan, Milan, Italy; ^7^European Foundation for the Care of Newborn Infants (EFCNI), Munich, Germany; ^8^Department of Pediatrics and Child Health, Nihon University School of Medicine, Tokyo, Japan; ^9^Department of Respiratory Medicine, Beijing Children's Hospital, Capital Medical University, Beijing, China; ^10^Department of Pediatrics, School of Medicine, University of Thessaly, Larissa, Greece; ^11^Faculty of Medicine, Institute for Life Sciences, University of Southampton, Southampton, United Kingdom; ^12^National Institute for Health Research (NIHR) Southampton Clinical Research Facility, NIHR Southampton Biomedical Research Centre, University Hospital Southampton NHS Foundation Trust, Southampton, United Kingdom; ^13^Pediatric and Infectious Diseases Unit, Bambino Gesù Children Hospital, Rome, Italy

**Keywords:** infant and child health, infant mortality, respiratory syncytial virus (RSV), RSV-acute lower respiratory illness, monoclonal antibody, infant hospitalization, infant immunization, respiratory disease

## Introduction: RSV burden concerns all infants

Respiratory syncytial virus (RSV) infects nearly all infants at least once by their second birthday (1). It spreads through coughs, sneezes, or close physical contact (2). RSV infections are associated with morbidity and mortality, ranging from mild upper respiratory illness to life threatening lower respiratory tract infections (LRTIs). More than 97% of RSV-attributable deaths occur in low- and middle-income countries (LMICs) (3), reflecting that healthcare infrastructure and resources in these countries is limited and may present issues in dealing with RSV disease burden.

Severe RSV infection is more likely during the first months of life (4). RSV causes a substantial burden for infants below 12 months of age. It is estimated that 12.9 million RSV LRTI episodes, 2.2 million RSV-associated hospitalizations and 66,300 RSV-attributable deaths occurred globally in 2019 (3). RSV also causes a significant outpatient burden worldwide, with RSV being associated with 21% of infants aged <24 months brought to the emergency department and 18% in pediatric practices according to one study (5).

RSV is a leading cause of hospitalization among infants in their first year of life, whether they are born during or before the RSV season (6). Severe RSV cases are difficult to predict. Although preterm infants or infants with co-morbidity have a higher risk of having a severe infection, ~80% of infants hospitalized with RSV are otherwise healthy, i.e., with no underlying medical conditions, and born at term (7). The percentage can be even higher in some countries. For example, it was reported in Japan that 98% of infants hospitalized for RSV were otherwise healthy (8). In addition, roughly 50% of all children hospitalized with RSV are born outside of the RSV season (9). Quantifying individual RSV risk is far more complicated than assessing population level RSV risk due to a number of interrelated risk factors (10). Multiple factors appear to put some children at higher risk of a severe RSV infection, such as having older siblings; having a parent who smokes; exposure to pollution; poor living conditions; living in the suburbs or large communities; siblings attending school or daycare; socio-economic status and a low level of parental education; maternal age; and a familial history of atopy.

RSV represents a significant economic burden. RSV infection increases the length of hospital stay and admissions to the intensive care department compared to non-RSV-related infections (11). RSV in premature and at-risk infants (i.e., those with congenital heart disease, chronic lung disease, neuromuscular impairment, immunodeficiency and Down's syndrome) incurs an individual economic burden that is comparatively higher than a healthy, full-term infant (12, 13). However, in an annual cohort of infants, RSV disease burden and associated costs (including hospitalization and outpatient visit costs) are created mostly by RSV in infants who were healthy prior to the acute RSV illness, due to the far higher number of hospitalizations observed in this infant population (14). Infant RSV infection also resulted in significant 5-year long-term healthcare-resource utilization impact (15). The etiological link between RSV infection and the development of asthma has long been debated. Many studies assume that RSV infection is a trigger of a pre-existing predisposition to asthma and can trigger further economic burden (3, 14, 16, 17).

RSV infections also place a significant burden and emotional impact on affected families and caregivers (18–20). Parents may feel powerless due to a lack of knowledge on RSV and its related complications. Given the overwhelming service needs during RSV season, which may overlap with other infectious disease's seasonality, healthcare staff are also impacted, often manifesting as stress and burnout (21).

## COVID-19 has impacted RSV circulation and reinforces the need for new prevention solutions

The COVID-19 pandemic has disrupted the epidemiology of many infectious diseases—including RSV. RSV usually peaks across the late autumn and winter in temperate countries (typically November to March) (22). While typically being more evenly distributed across the year in tropical regions, most countries in this region show a peak toward late summer (23). A minority of countries—almost all equatorial LMICs—report multiple peaks, or consistent RSV circulation year-round (23).

Non-pharmaceutical COVID-19 interventions such as social distancing reduced transmission, leading to an unusual reduction of RSV cases throughout 2020 in many countries (22). In 2021, following the removal of lockdown measures, uncommon resurgences during *spring and* summer were first reported in southern hemisphere countries such as Australia (24). Similar trends were then observed in some northern hemisphere countries, such as France, Spain, Germany and the UK (25).

The out-of-season peaks resulted in a significant disruption of healthcare systems and overwhelmed pediatric services due to RSV-related complications and potential hospital-acquired infections. In Germany, children aged 6 months to 1 year were hospitalized due to RSV across the summer of 2021 at a higher rate than they had been in previous years (26). Due to reduced circulation of RSV during the winter months of 2020, older infants and toddlers showed an increased risk of severe RSV-associated illness in 2021 (up to 5 years of age) (27).

## Future immunization solutions should be assessed with the objective of protecting all infants against RSV

All infants should ideally be protected during their first RSV season as long as the prevention solution provided is proven safe and cost-effective. The only currently available preventative measure is a mAb, palivizumab (AstraZeneca) which is indicated and approved in many high-income countries for some infants born preterm, and/or who have existing heart or lung disease and must be injected monthly throughout the RSV season (28).

Several active and passive immunization options are in late-stage development. These include new monoclonal antibodies and both pediatric and maternal vaccines. Clinical trials are ongoing (29). It is of utmost importance to have data on the efficacy and duration of protection for these new immunization options in order for Immunization Technical Advisory Groups to assess them properly, with the objective of protecting all infants against RSV and reducing the overall RSV burden.

**Figure 1:**
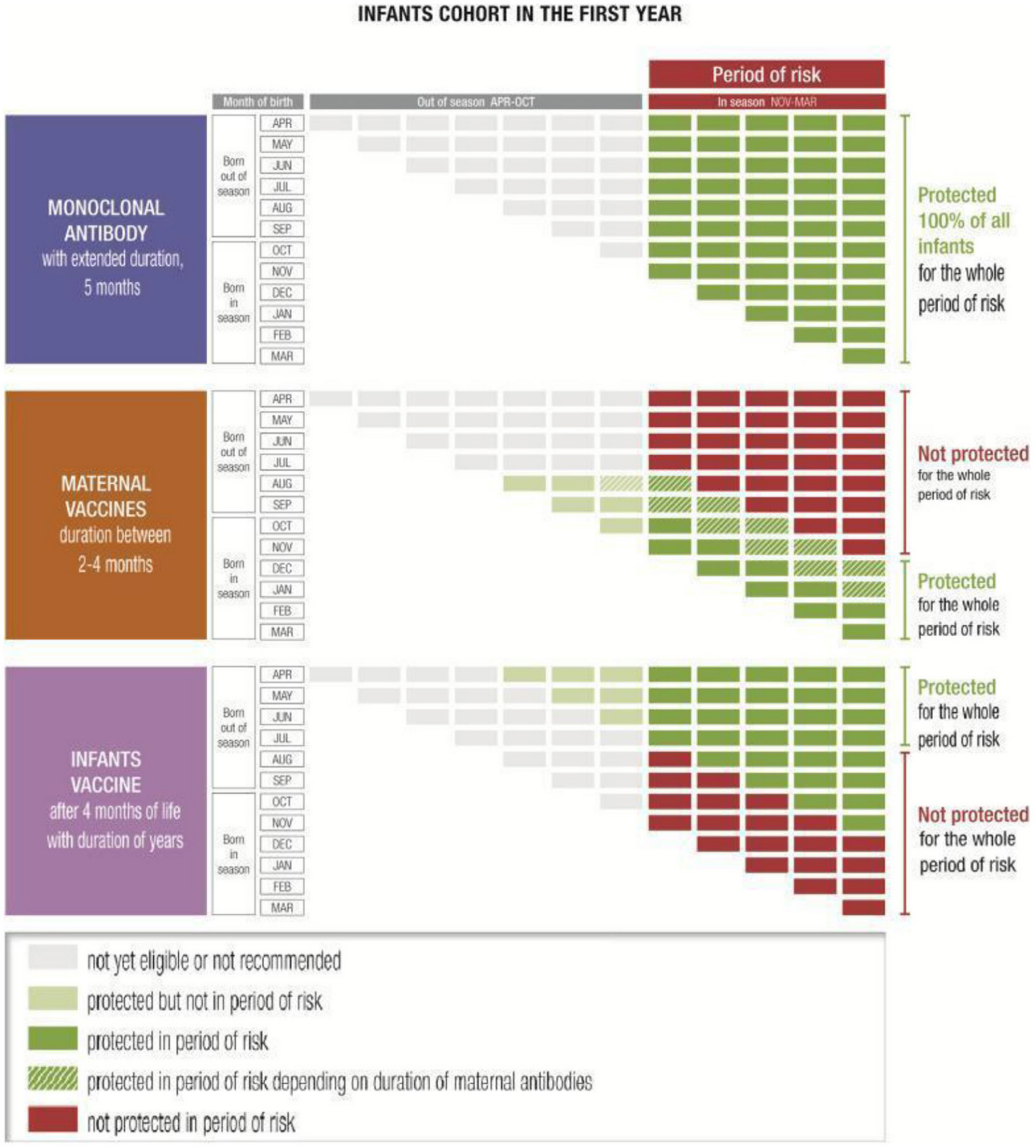
Examples of immunization coverage of infants through the first year of life against the November–March seasonality typical of temperate Northern hemisphere countries (35).

A long-acting mAb, which is likely to be the first licensed new preventative intervention, has shown promising results and there is a growing body of evidence suggesting it can protect all infants from RSV through their first RSV season with a single dose (30–34) (Figure 1). A baby born during or *just before* the RSV season should be immunized with a mAb as early as possible. The timing and location in which the immunization is administered is likely to differ depending on the country due to the differences in the postnatal practices and systems. Where possible, immunization should occur directly after birth, whether in the hospital, birth centers or in primary care settings (31, 35–37). Approaches that work within pre-existing immunization routine visits and structures are essential to ensuring uptake and to avoiding additional appointments for parents. The closest medical appointment to the start of the RSV season will be the optimal time for administration of the mAb to infants born before it (35). There is a degree of flexibility in the administration timing due to the rapid onset of protection with a mAb. Although acceptability of the RSV mAb palivizumab is currently high for infants at risk, it is unknown to what extent parents of healthy, full-term infants will accept long-acting mAbs against RSV (38–40).

Maternal vaccination is another option which might have great potential (41–43), even though the protected population might be more limited in cases of extreme prematurity where the infant may not receive the full benefit of antibody transfer (44), and for infants born out of season (35) (Figure 1). It may be that some health systems will offer parents a choice of prevention technologies: if a maternal vaccine is licensed, for babies born close to or in season, the mother may be offered the choice of receiving a vaccine during the third trimester or of the baby receiving a mAb shortly after birth. In countries and communities with poor maternal vaccine coverage, implementation of a RSV maternal vaccine could be challenging.

Both options (mAbs and maternal immunization) could be combined with RSV pediatric vaccines—which will likely be available at a much later date—to extend the duration of RSV protection throughout childhood (35) (Figure 1). This combined approach would grant immune coverage throughout the first months of life before immune system maturity is developed.

## Need for an increased awareness of the risk that RSV poses to all infants: A prerequisite for the assessment of new immunizations

Currently there is a general lack of education about the burden of RSV and especially about all infants' cohorts being at potential risk. Consequently, there is a lack of awareness about the differences and complementarities between future preventative solutions. Knowledge was even noted to be low among non-specialist medical practitioners (45).

Parents must be properly informed about the benefit and reassured about the safety profile of any preventative solution in appropriate language. Some of this education will be given by health practitioners including midwives, nurses, family doctors, pharmacists and pediatricians, depending on the country.

The current momentum of discussion surrounding infectious diseases must not be wasted. COVID-19 has sensitized policymakers to respiratory infectious diseases and their necessary prevention. In many countries, these are now high on the political agenda. Policymakers should be informed of the burden of RSV in all infants, the value of the different prevention solutions and how they can support public health objectives, and prioritize the assessment of new RSV immunization routine strategies. Overall, strategies to increase the uptake of immunization, as well as efforts to dispel harmful misinformation (46) must also be promoted.

Even though a lot of data is already available, there is still a need for improved national surveillance systems for respiratory viral infections. New epidemiological data will come from increased use of multiplex PCR and rapid tests in the wake of COVID-19. Policymakers and healthcare professionals should also consider collecting more data on rehospitalizations and outpatients visits due to the long-term sequelae of RSV infection in infancy, such as recurrent wheezing and asthma. These data may be important in aiding evidence-based decisions about prevention.

## Discussion

It is difficult to identify which infants will experience the most severe RSV illness and the vast majority of infants hospitalized with severe RSV are otherwise healthy and born at term. Beyond hospitalizations, RSV causes a significant outpatient burden. Policy recommendations to protect all infants against RSV should be discussed by Immunization Technical Advisory Groups. In each country, the development of recommendations will demand a careful assessment of the new RSV immunization programs and an analysis on the ease of implementation of these measures.

New immunization perspectives can protect more infants compared with the current standard of care. New long-acting mAbs are likely to be licensed first and can protect all infants, through administration at or close to birth for those infants born during the RSV season or alongside pre-scheduled immunization visits for those born before the season. That point of view is supported by additional publications released since the RSV Experts Group Event held in October 2021 (47, 48). Reducing RSV could relieve recurrent pressure on healthcare systems, leading to more efficient use of resources and contributing to the sustainability of healthcare systems.

The uptake of new RSV immunization strategies depends on the level of awareness of RSV among healthcare professionals, policymakers, and parents.

Although more is needed, awareness of RSV is improving. COVID-19 has played a huge role in this, with multiplex testing also checking for RSV cases in some countries. The data generated have made healthcare workers more aware of the volume of cases. The pandemic has also highlighted how crucial adequate immunization coverage is to maintaining both the health of the targeted population and the functioning of healthcare systems. As a consequence, many governments are now prioritizing the prevention of respiratory infectious diseases. As the burden of RSV is much higher than that of other pediatric infectious diseases, it is crucial to make the assessment of new RSV preventative measures for all infants a priority.

## Author's note

All authors participated in the RSV Experts Group Event held in October 2021. The discussions during this meeting were used as the basis of the content of the article.

## Author contributions

All authors listed have made a substantial, direct, and intellectual contribution to the work and approved it for publication.

## Funding

Sanofi and AstraZeneca facilitated and financially supported the RSV Experts Group Event, remuneration to some of the experts was agreed for the time spent for the meeting but the authors were not paid for writing the publication. Funding for the publication fees was provided by Sanofi and AstraZeneca. Hyderus CYF—was paid by Sanofi and AstraZeneca for the expert meeting organization, medical writing, and editorial support.

## Conflict of interest

LB has regular interaction with pharmaceutical and other industrial partners and has not received personal fees or other personal benefits and was the founding chairman of the ReSViNET Foundation. UMCU has received major funding (>€100,000 per industrial partner) for investigator initiated studies from AbbVie, MedImmune, AstraZeneca, Sanofi, Janssen, Pfizer, MSD and MeMed Diagnostics, has received major funding for the RSV GOLD study from the Bill and Melinda Gates Foundation, has received major funding as part of the public private partnership IMI-funded RESCEU and PROMISE projects with partners GSK, Novavax, Janssen, AstraZeneca, Pfizer and Sanofi, has received major funding by Julius Clinical for participating in clinical studies sponsored by MedImmune and Pfizer, and received minor funding (€1,000–25,000 per industrial partner) for consultation and invited lectures by AbbVie, MedImmune, Ablynx, Bavaria Nordic, MabXience, GSK, Novavax, Pfizer, Moderna, Astrazeneca, MSD, Sanofi, Genzyme, Janssen. EH has received speaking/advisory fees/travel support from Abbott (Astra Zeneca, Medimmune), Sanofi and Merck and has supported the German and the European (EFCNI) parents organization in the preparation of information material concerning RSV. UKSH has received a grant (no personal money for EH) to conduct a study (BRICE study) on the prevalence of RSV in Europe. SE reports Advisory Board Participation and Honoraria for Lectures: GSK, Janssen, Pfizer, Moderna, MSD, Qiagen, Sanofi, Genzyme, Janssen. SM reports a sponsorship agreement between SP and EFCNI. IM has received lecture fees from AstraZeneca K.K., MSD Co., Ltd., and Sanofi K.K. An honorarium was paid to SFs institution for his participation in the expert group by Sanofi but SF received no personal payments of any kind. SF has acted as clinical trial investigator on behalf of his hospital for GSK, Janssen (J&J), Regeneron and Medimmune (AstraZeneca) in the field of RSV vaccines and monoclonal antibodies but SF received no personal payment of any kind.

The remaining authors declare that the research was conducted in the absence of any commercial or financial relationships that could be construed as a potential conflict of interest.

## Publisher's note

All claims expressed in this article are solely those of the authors and do not necessarily represent those of their affiliated organizations, or those of the publisher, the editors and the reviewers. Any product that may be evaluated in this article, or claim that may be made by its manufacturer, is not guaranteed or endorsed by the publisher.

## Abbreviations

ALRI: Acute lower respiratory illness
mAbs: monoclonal antibodies
RSV: Respiratory syncytial virus
RSV-ALRI: RSV-associated acute lower respiratory illness
WHO: World Health Organization
ICU: Intensive Care Units
LMICs: Low- and middle-income countries.
